# *Marrubium eriocephalum* (Lamiaceae); a species new to the flora of Turkey, with contributions to its taxonomy

**DOI:** 10.3897/phytokeys.58.5890

**Published:** 2016-01-12

**Authors:** Mehmet Fırat

**Affiliations:** 1 Department of Biology, Faculty of Education, Yüzüncü Yıl University, TR-65080 Van, Turkey

**Keywords:** New record, Marrubium eriocephalum, Van, Turkey

## Abstract

*Marrubium eriocephalum* (Lamiaceae) is described as a new record for the Flora of Turkey (B9 Van). A detailed morphological description, photographs, distribution map, and pollen and nutlet morphology of this new record are given.

## Introduction

The genus *Marrubium* L. includes annual and perennial herbs. Although species of this genus are mainly distributed in the Irano-Turanian and Mediterranean phytogeographic regions, some members are naturalized in Australia and America. The genus comprises about 40 taxa (Akgül et al. 2008). In Turkey, the genus is represented by 21 taxa, of which 12 are endemic to Turkey (Cullen 1982, Davis et al. 1988, Ekim et al. 2000, Aytaç et al. 2012). This endemism rate (57%) shows that Turkey is an important centre of diversity for the genus. Bentham (1834, 1848) first revised the genus and divided it into sections *Lagopsis* and *Marrubium*. Later, many who studied the genus divided it into several sections (Briquet 1986, Boisser 1879, Seybold 1978); however, in Turkey, *Marrubium* was not divided into sections in recent treatments by Cullen (1982) and Akgül (2004).

Pollen grain features are taxonomically significant. A large number of studies on pollen morphology in Lamiaceae can be found in the literature (Erdtman 1945, Celenk et al. 2008, Moon et al. 2008a, 2008b, 2008c, Hassan et al. 2009, Aytac et al. 2012), The seed surfaces of Lamiaceae have been studied by many researchers (Husain et al. 1990; Demissew and Harley 1992, Marin et al. 1996, Budantsev and Lobova 1997, Jamzad et al. 2000, Kaya et al. 2009, Kahraman et al. 2009, 2010, Akgül et al. 2008). In Lamiaceae pollen grains are reticulate, tricolpate or hexacolpate. (Erdtman 1945).

Erdtman (1945) studied *Marrubium* pollen grains. Abu-Asab and Cantino (1994) studied the pollen grains of Lamiaceae species, including the Turkish *Marrubium*. *Marrubium* pollen grains are tricolpate, radially symmetric and isopolar, and are prolate spheroidal and oblate spheroidal in shape (Erdtman 1969). There is no operculum. The aperture membranes are generally psilate or rarely granulate. The exine is tectate.

A detailed pollen morphological study of Turkish *Marrubium* species was undertaken by Akgül et al. (2008), where the pollen features of 19 Turkish taxa were examined and the pollen grains were divided into 3 groups: 1) the exine is psilate-perforate, psilate-foveolate and the pollen shape is prolate spheroidal, oblate-spheroidal; 2) the exine is granulate-perforate; and 3) the exine is reticulate, rugulate-reticulate and the pollen shape is prolate-spheroidal.

Lamiaceae nutlet surfaces are also taxonomically significant; they have different sizes and colours, so the nutlet morphology is used not only between the genera but also between subsections and subspecies (Husain et al. 1990). *Marrubium* nutlet surface morphology also has a systematic significance (Brochmann 1992, Hedge 1992).

## Materials and methods

**Figure 1.**
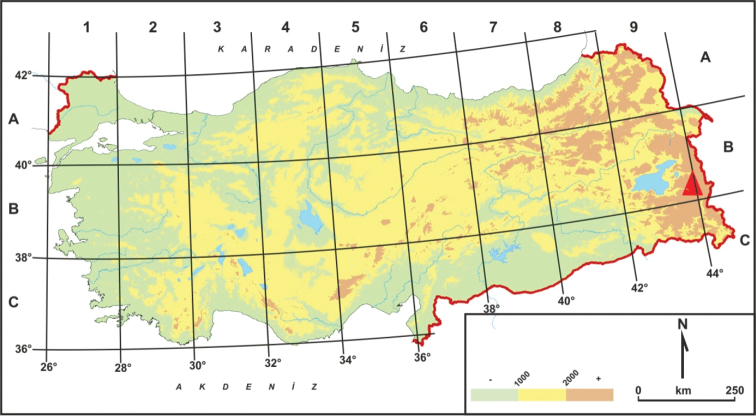
Geographical distribution of *Marrubium eriocephalum* Maroofi (▲) in Turkey.

*Marrubium eriocephalum* Seybold, was collected in İspiriz Mountains (Fig. 1), Van in October 2013-2014. Cullen (1982), and Güner (2000) did not record this species in Turkey. Thus this collection constitutes a new record for the Turkey flora: Cullen (1982), Boissier (1859), Huber-Morath (1978), Seybold (1978), Özhatay et al. (2009), Özhatay and Kültür (2011), Akgül (2012) only recording *Marrubium eriocephalum* from South Kurdistan region of Iraq. Collected materials were deposited at the Herbarium of Yüzüncü Yıl University Science Faculty (VANF).

*Marrubium eriocephalum* pollen grains were studied using LM and SEM. The voucher specimens are listed in (Table 1). For the LM, the pollen grains were first treated with 70% alcohol and allowed to evaporate, and then embedded in glycerine jelly (Wodehouse 1935). The polar axis (P), equatorial axis (E), colpus length (Clt), colpus width (Clt), exine thickness (Ex), intine thickness (I), and apocolpium diameter (Ap) were measured from at least 30 fully developed grains per sample under an Olympus BX21 microscope (1000×). For the SEM analyses, pollen grains obtained from each specimen were transferred onto stubs and coated with gold (JEOL JSM 7001-F). The methods of Henderson et al. (1968), Faegri and Iversen (1989), and Punt et al. (2007) were those mainly followed.

**Table 1.** Pollen morphology of *Marrubium eriocephalum* Unit is μm.

Taxon	Polar axis (μm)			Equatorial axis (μm)			P/E ratio Shape	Exine (μm)	Intine (μm)	Ornamentation
	min	max	ort.	min	Max	ort				
*Marrubium eriocephalum*	24.96	31.20	27.49	29.12	33.28	30.75	0.89 oblate-spheroidal	1.91	1.14	Psilate-reticulate

The seed morphology of this new record, *Marrubium eriocephalum*, was studied using SEM according to the methods of Murley (1951) and Koul et al. (2000).

## Description

### Marrubium eriocephalum

Taxon classificationPlantaeLamialesLamiaceae

#### Type.

**Figure 2.**
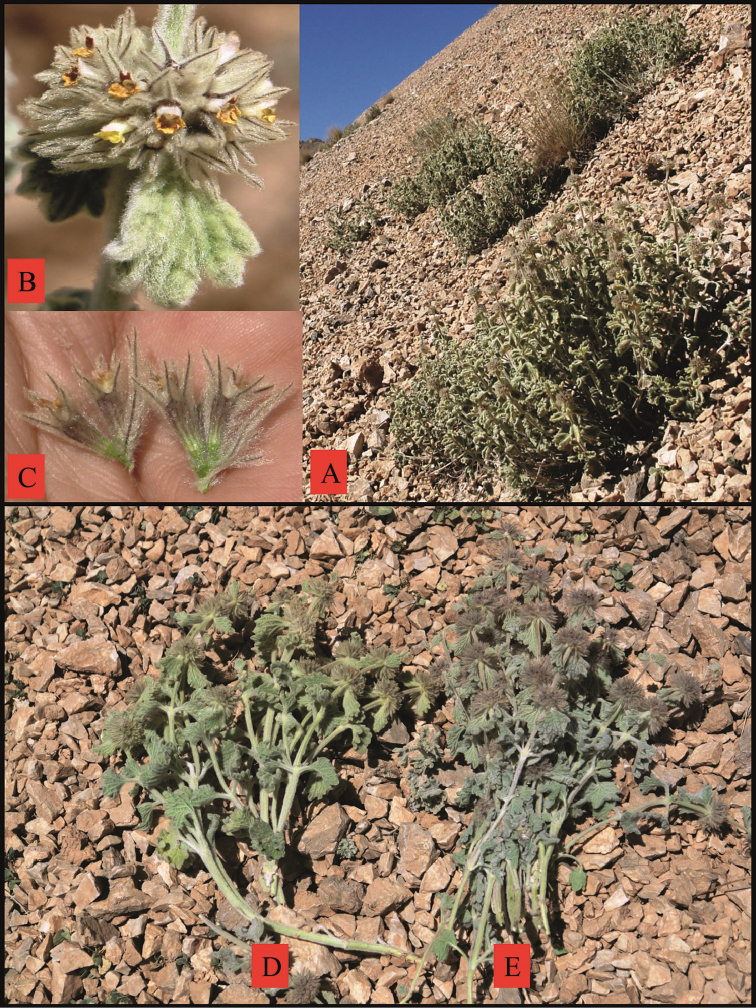
*Marrubium eriocephalum* **A** habit and habitat **B** flowers **C** calx and bracteoles **D** yellowish form **E** greyish form.

**Figure 3.**
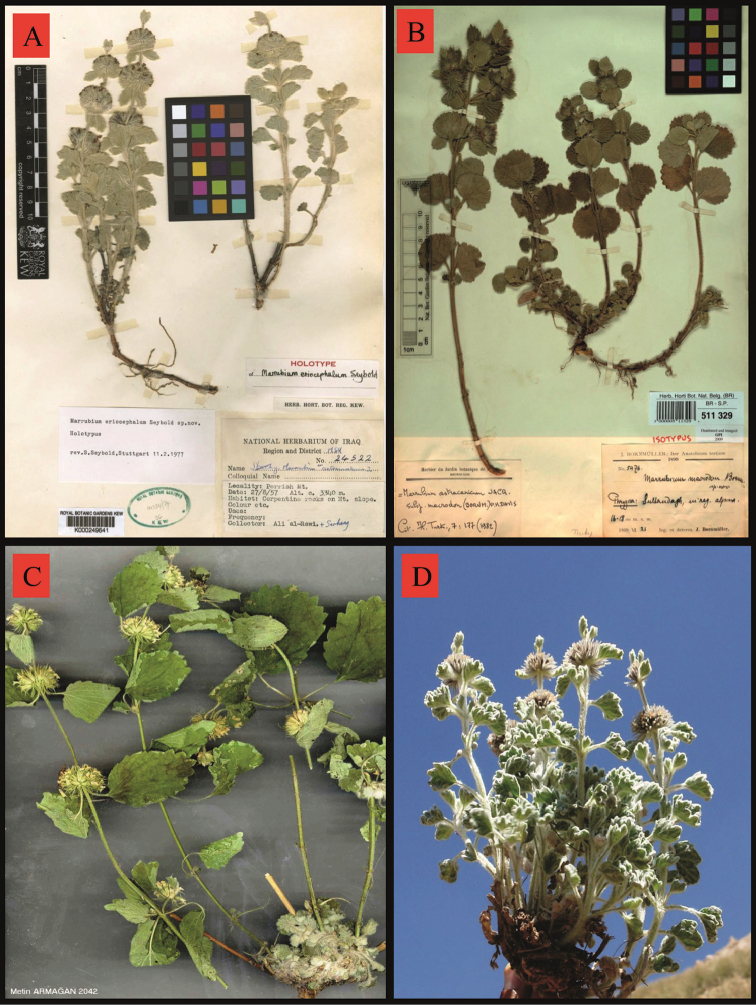
**A** *Marrubium eriocephalum* holotype Ali-al-Rawi&Serhang 24522 K **B** *Marrubium astracanicum* Isotype Bornmueller 5476 K **C** *Marrubium vanense* Metin Armağan 2042 VANF **D** *Marrubium eriocephalum* habit (İspiriz Mountaiuns-fresh).

Iraq: Perrish, 3340 m, 27.08.1957, Ali-al-Rawi & Serhang 24522, (holotypus K; http://specimens.kew.org/herbarium/K000249641). (Figs 2–3, Table 2).

**Table 2.** A comparison of selected morphological characters of *Marrubium eriocephalum* between descriptions given in the Type specimen and in the present study, *Marrubium vanense* and *Marrubium astracanicum*.

Morphological characters	*Marrubium eriocephalum* (Type specimen)	*Marrubium eriocephalum* (The findings of present study)	*Marrubium vanense*	*Marrubium astracanicum*
**Stems**	suffrutescent, multi stemmed, 15–25 cm long	ascending erect, branched, 15–50 cm long	erect, 50 cm or more	erect, somewhat branched, (15-)25–50(-70) em
**Indumentum**	densely white velutinous hairy	yellowish to greyish, densely white lanate hairy	longer and shorter simple hair	densely stellate-pilose, glabrescent
**Basal leaves**	rotund, cuneata, irregular crenate, up to 20× 20 mm	elliptic to rotund, irregularly or regularly crenate to serrate,lamina 10–22×21–35 mm	eliptic to ovate, crenate	elliptic-obovate, crenate
**Cauline leaves**	leaves rounded	petiolate, flabellat, lunat-rotund, irregularly or regularly crenate-serrate	Petiolate, ± orbicular or flabellate, irregularly and coarsely crenate	long-petiolate, orbi cular to elliptic-obovate, deeply and irregularly crenate-serrate
**Leaves petiol**	up to 15 mm long	8–20 mm long	15–30 mm long	5–15 mm long
**Verticillasters**	23–25 mm diameter, 20–30 flowers	18–27 mm diameter, 15–35 flowers	15–25 mm diameter, 15–25 flowers	10–15 mm, 15–20 flowers
**Bracteoles**	subulate, 8–10 mm long, distinctly hairy with long white pilose	subulate, 10–14 mm long as long as long calyx teeth, densely spreading stellate villous to lanate hairy	subulate, 4–5 mm long, as long as calyx tube	Subulate, 5.5–9 mm long, as long as calyx teeth
**Calyx**	calyx tube 4,5 mm, densly white glandular pilose hairy, purple; calyx teeth 5, straight or spreading, purple, 2,5 mm long, subequal, apical pilose hairy	calyx tube 5–7.5 mm purplish to greenish, tubular, 5 subequal teeth and teeth 2.5–5.5 mm long, erect, long stellate hairy with short or sessile glandular hairs	Calyx tube 4.5–5 mm, with stellate hairs; teeth 5 or rarely 6, somewhat unequal, 22.5 mm, straight, erect or slightly spreading, covered with stellate hairs for 1/2-2/3 of their length	Calyx tube 4-5 mm, sparsely to densely spreading pilose with stellate hairs with elongate central branches. Teeth 1-4 mm, usually dark purple, straight, erect, stellate-pilose for c. 2/3 of their length
**Corolla**	purple, 6–8 mm, galea 1,5 mm, third part bifid, galea 3–4 mm with lip	yellowish sometimes purplish on upper lip, yellowish within lip, 7–11 mm long, upper lip (galea) 0.5–1 mm, 2-lipped; upper lip straight, bifid; lower lip 3-lobed, middle lop of the lower lip 3–4 mm wide, densely long stellate hairy, lower part of the corolla tube sometimes with glabrous and/or a few sessile glandular hairy	yellowish-white, densely lanate with stellate hairs outside, upper lip glabrous inside	lavender, mauve or purple, 10-14 mm, densely stellate pubescent outside, ±glabrous within upper lip.
**Nutlets**	unkonwn	brownish to greenish, oblong, 2.5–3.4 mm long×1.1–2.1 mm wide.	oblong, brown-dark brown, 2 mm long×1.3 mm wide.	oblong, brown, 1.9 mm long×1.1 mm wide.

Ascending erect, branched, perennial herb. Stems 15–50 cm, yellowish to greyish, densely white lanate hairy. Basal leaves elliptic to rotund, petiolate, petiole 8–20 mm, lamina 10–22×21–35 mm, irregularly or regularly crenate to serrate, densely lanate with stellate hairs. Cauline leaves petiolate, flabellate, lunate or rotund, irregularly or regularly crenate-serrate, and densely lanate with stellate hairs. Verticillasters, 1–3(5), globular 18–27 mm diameter, 15–35 flowered. Bracteoles subulate, as long as long calyx teeth, densely spreading stellate villous to lanate hairy, 10–14 mm. Calyx purplish to greenish, tubular, tube 5–7.5 mm long, 5 -toothed with teeth subequal 2.5–5.5 mm long, erect, long stellate hairy with short or sessile glandular hairs. Corolla yellowish sometimes purplish on upper lip, yellowish within lip, 7–11 mm long, 2-lipped; upper lip straight, bifid 0.5–1 mm long; lower lip 3-lobed, middle lobe of the lower lip 3–4 mm wide, densely long stellate hairy, lower part of the corolla tube sometimes with glabrous and/or a few sessile glandular hairy. Nutlets brownish to greenish, oblong, 2.5–3.4 ×1.1–2.10 mm, verrucate. Pollen grains isopolar, tricolpate, oblate (P/E 0.89), polar axis (P) 27.49 μm, equatorial axis (E) 30.75 μm, amb triangular, exine 1.91 μm, the colpus membrane granulate, exine psilate- reticulate.

#### Examined material.

Turkey. B9 Van; Başkale, İspiriz Mountains West, Serpantine rocks, 3259 m, 38°04'17"N, 43°56'23"E, 11.09.2013, *M. Fırat* 30289 & E. *Hamzaoğlu* (in flower), VANF; ibid *M. Fırat* 30335 (in fruit) VANF; B9 Van; Başkale, İspiriz Mountains East, Mobile limestone screes, near serpentine rocks, 3419 m, 38°05'04"N, 43°57'26"E, 15.09.2014, *M. Fırat* 31010 (in flower), VANF.

#### Habitat.

Mobile limestone screes, near serpentine rocks, 3200-3500 m, in very sparse vegetation.

#### Phenology.

Flowering and fruiting times from August-October.

#### Distribution in Turkey.

Van province.

**General distribution.** Kurdistan region of Iraq, Turkey.

#### Associated with.

*Allium oreophilum* C.A.Mey., *Didymorphysa aucheri* Boiss., Jurinella moschus (Hablitz) Bobrov subsp. moschus, *Heracleum* sp.

#### Vernacular name.

In Van province, indigenous people use the name “Bizbizok” for *Marrubium* (Fırat 2013).

## Results

### Palynological investigation

The pollen grains were measured and photos were taken with an Olympus BX21 light microscope. The pollen type, exin surface ornamentation, and SEM microphotography were recorded. The seed sizes were measured and the surface ornamentation was scrutinized with SEM microphotography.

**Figure 4.**
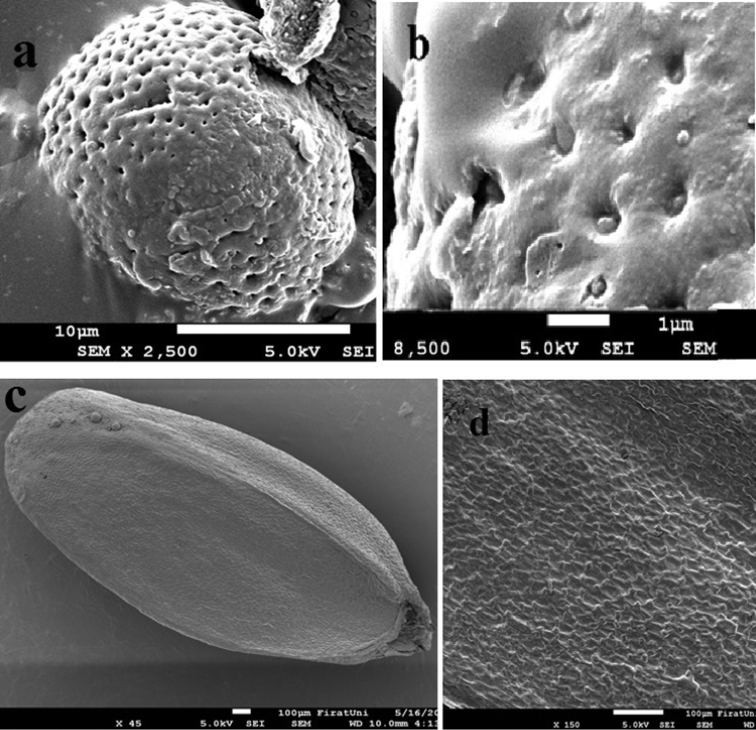
SEM photos of the pollen grains and seed coat of *Marrubium eriocephalum*; **a** equatorial view (×2500) **b** detail of pollen grains (×8500) **c** general shape of seed coat (×45) **d** seed coat surface (×150).

The *Marrubium* pollen grain measurements are given in (Table 1). The pollen grains were isopolar, tricolpate, and oblate (P/E 0.89), with a polar axis (P) of 27.49 μm and an equatorial axis (E) of 30.75 μm. The amb was triangular, the exine was 1.91 μm thick, the colpus membrane was granulate, and the exine was psilate- reticulate (Table 1, Fig. 4)

### Nutlet morphology

The nutlet morphology of *Marrubium eriocephalum* was studied herein for the first time. Nutlet of the species were large, brown, and eliptic. The average nutlet size was 2.5–3.48 × 1.18–2.10 mm. There were polygonal cells in the nutlet epicarp and the ornamentation was verrucate.

## Discussion

The *Marrubium eriocephalum* was first collected from the Kurdistan region of Iraq by Ali-al-Rawi & Serhang, and described as a new species by Seybold (1978). Differences based on observations and measurements during 2-year field trips and herbarium studies, between type specimens and our material, are shown in Table 2. Specifically, Seybold described the species as having purple flowers; however, our findings showed that the species has yellowish flowers with purple upper lips. We believe that this error was due to the fact that the observations by Seybold were based solely on herbarium material. However, the author of *Marrubium eriocephalum*, Seybold (1978) claimed that *Marrubium astracanicum* is a species close to *Marrubium eriocephalum*, yet our findings showed that *Marrubium vanense* Hub.-Mor. is closer to *Marrubium eriocephalum* according to Flora of Turkey (Table 1, Fig. 3) in stem height, bracteole length and leaf indumentum.

Our palynological findings were compared with those of the study of Akgül et al. (2008) and according to their classification, *Marrubium eriocephalum* is classified as the third type. Due to its oblate-spheroidal pollen grains, psilate-reticulate ornamentation, and tricolpate aperture, our pollen showed similarities with *Marrubium vanense* and *Marrubium catariifolium* Desr. However, *Marrubium catariifolium* is different from *Marrubium eriocephalum* in being an annual, with white flowers in 5-8 flowered verticilasters Cullen (1982).

In Flora of Turkey, the nutlet features of *Marrubium vanense* and *Marrubium catariifolium* are described. Both species have oblong, brown-dark brown nutlet with verrucate ornamentation. Our investigation showed similarities with the nutlet features of *Marrubium vanense*, *Marrubium catariifolium*, and *Marrubium eriocephalum*, which are oblong, brown, and verrucate in ornamentation.

### Key to closely related *Marrubium* species in Turkey

**Table:** 

1	Corolla mauve to purple	***Marrubium astracanicum***
–	Corolla white, cream or yellowish, rarely pinksh	**2**
2	Verticillasters 1-flowered, disposed in long ‘spikes’	***Marrubium depauperatum***
–	Verticillasters several-flowered, not disposed in ‘spikes’	**3**
3	Plants widely and divaricately branched; calyx teeth stellate- pubescent to apex	***Marrubium peregrinum***
–	Plants unbranched, or with few, erect branches; calyx teeth usually with upper 1/3 or 1/2 glabrous	**4**
4	Stems 50 cm or more; calyx tube greenish; leaves with simple hairs above	***Marrubium vanense***
–	Stems 50 cm or less; calyx tube purplish to greenish; leaves with densely stellate lanate above	***Marrubium eriocephalum***

## Supplementary Material

XML Treatment for Marrubium eriocephalum
